# Ultrasound-guided one-point puncture lumbosacral plexus block combined with laryngeal mask airway general anesthesia for thigh amputation in a patient with mucopolysaccharidosis: a case report

**DOI:** 10.3389/fmed.2026.1790821

**Published:** 2026-03-12

**Authors:** Shengrun Gao, Junfeng Dong, Chengjie Gao, Xiaohong Zhao

**Affiliations:** 1 Department of Anesthesiology, The 960th Hospital of PLA, Jinan, China; 2 Department of General Surgery, The 960th Hospital of PLA, Jinan, China

**Keywords:** difficult airway, lumbosacral plexus block, mucopolysaccharidosis, multimodal analgesia, one-point puncture, regional anesthesia

## Abstract

Mucopolysaccharidosis (MPS) is a group of rare inherited lysosomal storage disorders caused by deficiencies of specific enzymes, leading to abnormal accumulation of glycosaminoglycans in tissues throughout the body. It is often associated with a difficult airway, cervical spine instability, restrictive pulmonary dysfunction, and cardiovascular pathologies, which significantly increase perioperative risks. This manuscript reports a case of a 36-year-old male MPS patient (clinical phenotype highly suggestive of type IV) who underwent right mid-to-upper thigh amputation for a pathological fracture of the right femur. Given his potential difficult airway (Mallampati class III, thyromental distance <6 cm), mild kyphosis, and restrictive pulmonary dysfunction, the anesthetic plan consisted of an ultrasound-guided one-point puncture, dual-target lumbosacral plexus block (35 mL of 0.15% ropivacaine) combined with supraglottic airway (laryngeal mask airway, LMA) under general anesthesia. The block successfully provided an L2-S3 sensory level and served as the basis for postoperative multimodal analgesia. The surgery lasted 2 h with 500 mL blood loss, requiring 2 units of red blood cells. Hemodynamics remained stable, and no additional muscle relaxants were administered. The patient regained consciousness and the LMA was removed 10 min postoperatively. The Numerical Rating Scale (NRS) pain scores at 2, 6, 12, and 24 h postoperatively were 2, 3, 2, and 1, respectively. Functional exercise began on postoperative day 1 without major complications. The patient was discharged 15 days after surgery and was hospitalized for a total of 18 days. With strict patient selection and thorough preparation of emergency airway protocols, combining an LMA with an ultrasound-guided one-point puncture lumbosacral plexus block can be a safe and feasible individualized anesthetic strategy for lower limb proximal surgery in MPS patients. This approach helps avoid intubation risks, reduces opioid consumption, and promotes early recovery. However, due to the considerable difficulty of airway management in patients with MPS, elective surgery requires multidisciplinary consultation and comprehensive airway assessment to ensure perioperative safety.

## 1 Introduction

Mucopolysaccharidosis (MPS) is a rare inherited metabolic disorder caused by lysosomal enzyme deficiencies, leading to the progressive accumulation of glycosaminoglycans in tissues throughout the body, which can involve multiple organ systems. Common clinical manifestations include coarse facial features, joint contractures, spinal deformities, restrictive pulmonary dysfunction, cardiac valvular lesions, and upper airway structural abnormalities. MPS is clinically classified into types I, II, III, IV, VI, VII, and IX. Among these, types I, II, and IV are often associated with difficult airways and cervical spine instability, posing multiple challenges for perioperative anesthesia management (1–3). While traditional tracheal intubation general anesthesia in MPS patients ensures ventilatory safety, laryngoscopy maneuvers may induce excessive cervical spine extension, carrying a risk of spinal cord injury. Concurrently, subglottic stenosis, macroglossia, and thickening of pharyngeal soft tissues significantly increase the risk of failed intubation, potentially necessitating emergent surgical airway establishment (2, 4). Epidural or subarachnoid block is suitable for lower limb surgery anesthesia. However, MPS patients may have severe spinal deformities, osteoporosis, vertebral anomalies, and anatomical variations in the epidural space, potentially leading to block failure or even nerve injury, thereby increasing the risks associated with neuraxial anesthesia (5–7). Ultrasound-guided, visual peripheral nerve blockade techniques demonstrate considerable value in the surgical anesthesia of MPS patients (8). Combined lumbosacral plexus block effectively covers the innervation areas of the femoral, obturator, and sciatic nerves, providing comprehensive sensory and motor blockade (9). In recent years, Gong et al. (10) proposed a one-point puncture, dual-path injection technique for combined lumbosacral plexus block in the supine position, which achieves both lumbar and sacral plexus blocks through a single puncture. This technique significantly reduces the number of punctures and patient repositioning, showing good clinical utility. However, there is scarce literature reporting the application of this technique in MPS patients, and its feasibility, safety, and clinical value require further validation. This manuscript reports for the first time the anesthetic management process of an adult MPS patient undergoing right thigh amputation under ultrasound-guided one-point puncture combined lumbosacral plexus block supplemented with laryngeal mask general anesthesia. The aim is to provide a safe and effective anesthetic strategy for MPS patients.

## 2 Case presentation

### 2.1 Patient information

**Figure 1:**
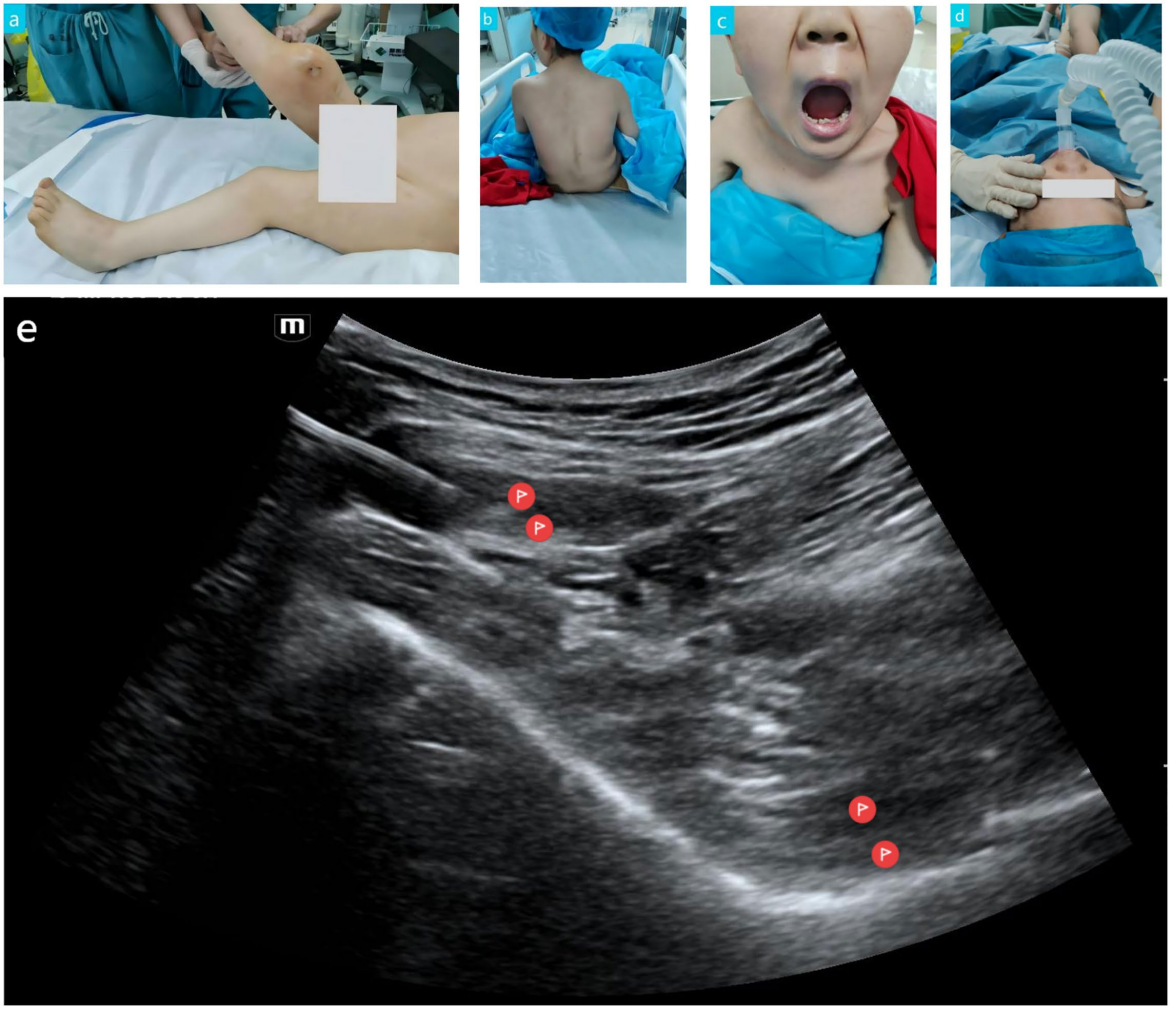
Perioperative image data of the MPS patient. **(a)** Pathological fracture of the right distal femur. **(b)** Mild spinal deformity. **(c)** Mouth opening and Mallampati classification. **(d)** Insertion of a size 2.5 laryngeal mask airway. **(e)** Needle insertion site for lumbar-sacral plexus nerve block.

The patient was a 36-year-old male with a height of 100 cm and a weight of 25 kg. He was transferred to the 960th Hospital of PLA on May 30, 2025, due to a “pathological fracture of the right femur” (Figure 1a). His medical history confirmed a diagnosis of MPS, with the specific subtype pending confirmation by further genetic testing. However, physical examination revealed coarse facial features, extremely short stature, severely restricted joint mobility, and mild spinal deformity (Figure 1b). The clinical phenotype was highly suggestive of MPS type IV (Morquio syndrome), although MPS type I or II could not be entirely ruled out. Chest CT showed patency of the trachea and main bronchi. Postoperative pathological diagnosis: “(Right femur) High-grade soft tissue sarcoma. Combined with immunohistochemical staining results, it is consistent with pleomorphic undifferentiated sarcoma.”

### 2.2 Preoperative assessment

The preoperative assessment indicated a potentially difficult airway and cervical spine instability. Mallampati classification was Grade III (Figure 1c), indicating a risk of difficult tracheal intubation. Additionally, the patient had a mild spinal deformity, constituting a contraindication for neuraxial anesthesia. Furthermore, the patient presented with restrictive pulmonary dysfunction, necessitating close monitoring of lung function protection and respiratory changes. Electrocardiogram (ECG) and cardiac ultrasound revealed no significant active cardiac valvular lesions, although the potential adverse effects of MPS on the heart could not be ignored. Therefore, an individualized anesthetic strategy was adopted for this patient: ultrasound-guided one-point puncture combined lumbosacral plexus block supplemented with laryngeal mask general anesthesia. This approach aimed to reduce anesthetic risk and optimize perioperative management, with preparations in place for potential poor laryngeal mask ventilation and difficult airway scenarios.

### 2.3 Anesthetic decision and implementation

#### 2.3.1 Airway management

Following standard ASA monitoring (ECG, arterial blood pressure, SpO₂, EtCO₂) and adequate preoxygenation, induction was achieved with intravenous injections of remimazolam besylate 3 mg, etomidate 5 mg, sufentanil citrate 15 μg, and cisatracurium 5 mg. After satisfactory induction, an experienced anesthesiologist successfully inserted a size 2.5 laryngeal mask (Figure 1d). Ventilation was smooth, with peak airway pressure maintained around 12 cmH₂O. The position was good with no air leak.

#### 2.3.2 Regional anesthesia

With the patient maintained in the supine position, a low-frequency curvilinear ultrasound probe was placed transversely at the level of the anterior superior iliac spine and slowly moved caudally until the anterior inferior iliac spine, iliopsoas muscle, transversus abdominis muscle, and internal oblique muscle were clearly visualized (Figure 1e). Using an in-plane approach, the needle was advanced from lateral to medial, passing through the abdominal wall muscles and the iliopsoas muscle. The needle tip was positioned deep to the iliopsoas (sacral plexus), and after negative aspiration for blood, 20 mL of 0.15% ropivacaine was injected. The needle was then withdrawn subcutaneously, its direction was adjusted, and the tip was repositioned into the fascial plane between the transversus abdominis and internal oblique muscles. It was advanced anteriorly to the area anterior to the anterior inferior iliac spine (where lumbar plexus branches course). Following another negative aspiration for blood, 15 mL of 0.15% ropivacaine was injected. This method effectively achieved combined lumbar and sacral plexus blockade with a single puncture and two target injections while the patient remained supine. This avoided the potential risks associated with repositioning in a patient with spinal deformity and provided comprehensive anesthesia coverage for proximal lower limb surgery.

### 2.4 Intraoperative management

Mechanical ventilation was maintained via the laryngeal mask throughout the surgery. General anesthesia was maintained with a propofol infusion at 4 mg/kg/h, a remifentanil infusion at 20 μg/kg/h, and inhalation of 1% sevoflurane. Bispectral index (BIS) monitoring remained between 40 and 60, indicating satisfactory anesthetic depth. The patient’s vital signs remained stable intraoperatively, with no need for vasoactive support. The surgery lasted 2 h and proceeded smoothly. Intraoperative blood loss was approximately 500 mL, requiring transfusion of 2 units of red blood cells and 500 mL of crystalloid fluid. Additionally, a fluid warmer was used for blood and fluid transfusions.

### 2.5 Postoperative outcome

The patient regained spontaneous respiration 10 min after surgery, was conscious, and the laryngeal mask was removed. He was then transferred to the Post-Anesthesia Care Unit (PACU). A multimodal analgesia regimen was implemented postoperatively: based on the lumbosacral plexus block, it was combined with Patient-Controlled Intravenous Analgesia (PCIA; formula: sufentanil 2 μg/kg diluted with normal saline to 100 mL, with a background infusion of 2 mL/h, a PCA bolus of 0.5 mL, and a lockout interval of 15 min). The Numerical Rating Scale (NRS) pain scores at 2, 6, 12, and 24 h postoperatively were 2, 3, 2, and 1, respectively. On postoperative day 1, the patient began bed-based functional exercises under the guidance of a physiotherapist. During the hospital stay, there were no adverse events such as laryngeal mask-related complications (e.g., sore throat, aspiration), local anesthetic toxicity, nerve injury, or deep vein thrombosis. The patient’s general condition was good. The patient was discharged 15 days after surgery and was hospitalized for a total of 18 days.

## 3 Discussion

The successful outcome of this surgery hinged on a thorough understanding of the pathophysiological characteristics of MPS and the individualized combined anesthetic strategy formulated accordingly.

### 3.1 MPS subtyping, diagnosis, and risk assessment

Although genetic confirmation was not obtained, the patient’s coarse facial features, joint contractures, kyphosis, normal intelligence, and survival into adulthood strongly suggested MPS type IV, while types I and II could not be ruled out. These three subtypes share features including upper airway narrowing, atlantoaxial instability, and cardiac valvulopathy (1–3, 7). Therefore, performing neuraxial anesthesia in such patients presents technical challenges and difficulties in balancing patient safety with comfort. Additionally, regarding airway assessment, it is important to note that enzyme replacement therapy and hematopoietic stem cell transplantation are effective treatments for MPS patients, but their impacts on the airway differ. The former can reverse structural airway lesions through early intervention, significantly reducing airway management risks, whereas the latter, while stabilizing disease progression, cannot repair established anatomical abnormalities such as laryngeal stenosis, making its airway management risks non-negligible (11, 12). In this particular case, the patient had acceptable daily living abilities and had not undergone the aforementioned treatments. Preoperative chest CT showed patent trachea and main bronchi. The Mallampati classification was grade III (Figure 1c), indicating that the risk of a difficult airway could not be ruled out. Airway abnormalities in adult MPS patients pose significant challenges for anesthesia management, and traditional bedside airway assessment has many limitations. A study by Mayhew demonstrated that integrating otolaryngological endoscopy, CT 3D reconstruction, virtual endoscopy, virtual reality simulation, and applying the Salford Adult MPS Airway Score to quantify overall risk is effective (13). For ultra-high-risk patients with a history of multiple intubation failures, using 3D-printed physical airway models for high-fidelity simulation training can successfully facilitate safe and controlled difficult airway management, significantly reducing perioperative airway complications. For elective surgeries, these assessment methods should be employed whenever possible to accurately evaluate the airway status of MPS patients and achieve controllable airway management risks.

### 3.2 Rationale for laryngeal mask airway selection

The use of an LMA circumvented the risks associated with tracheal intubation and direct laryngoscopy, which could potentially cause excessive cervical spine extension and subsequent spinal cord injury. In this case, preoperative chest CT confirmed a patent airway. The LMA provided effective ventilation with low peak pressures, demonstrating its feasibility in MPS patients. This suggests that, in carefully selected cases, an LMA can be a viable alternative, though vigilance is required for the risk of ventilation failure due to subglottic stenosis. Other studies confirm that adult MPS patients frequently present with valvular thickening leading to stenosis or regurgitation, cardiomyopathy, and coronary artery disease, increasing the risk of perioperative adverse cardiac events (1, 3). The LMA exerts minimal hemodynamic impact, making it particularly suitable for patients with potentially reduced cardiac functional reserve (14). It should be clarified that the laryngeal mask airway may reduce the risk of excessive neck movement, but it should not be considered the primary airway management strategy for the majority of MPS patients, especially for those with cervical instability. For such patients, video laryngoscopy remains the preferred method for tracheal intubation (5).

### 3.3 Technical advantages of ultrasound-guided one-point puncture lumbosacral plexus block

Patients with lower limb fractures often cannot tolerate the lateral decubitus position. This case employed a supine position, allowing the one-point puncture, dual-path injection technique without repositioning. This avoided the pain associated with movement and the challenges of lateral positioning in patients with spinal deformities. Compared to the traditional two-point puncture technique, this method is less traumatic, aligning with the pathological features of osteoporosis and joint stiffness in MPS patients. It provides more comprehensive blockade than single nerve blocks, offering ideal analgesia for mid-to-high thigh amputation.

### 3.4 Multimodal analgesia for reducing opioid use and promoting early recovery

General anesthesia ensured patient comfort and a quiet surgical field, while effective regional blockade attenuated the stress response to nociceptive stimuli (15). This synergistic effect significantly reduced the requirement for opioids, thereby lowering the incidence of postoperative side effects such as nausea, vomiting, and respiratory depression. This approach facilitates early mobilization, reducing the risks of pulmonary infection and deep vein thrombosis (16).

## 4 Conclusion

For MPS patients undergoing proximal lower limb surgery, ultrasound-guided one-point puncture lumbosacral plexus block combined with LMA-based general anesthesia represents a safe and feasible anesthetic management model. This is contingent upon a thorough assessment of airway anatomy, strict patient selection based on indications, and comprehensive preparation for emergencies. This strategy balances airway safety, hemodynamic stability, and effective analgesia, embodying the principles of individualized and precise anesthetic management. It provides a valuable practical reference for managing such rare and complex high-risk patients. It is particularly important to note that the success of airway management in this case was inseparable from the extensive clinical experience of the anesthesia team. For younger anesthesiologists, when managing MPS patients, comprehensive and systematic preoperative assessment should be conducted to ensure the accuracy of airway evaluation and the safety of anesthesia management.

## Data Availability

The original contributions presented in the study are included in the article/supplementary material, further inquiries can be directed to the corresponding authors.
